# Metabolic Syndrome and Neuroprotection

**DOI:** 10.3389/fnins.2018.00196

**Published:** 2018-04-20

**Authors:** Melisa Etchegoyen, Mariana H. Nobile, Francisco Baez, Barbara Posesorski, Julian González, Néstor Lago, José Milei, Matilde Otero-Losada

**Affiliations:** ^1^Institute of Cardiological Research, School of Medicine, University of Buenos Aires, Buenos Aires, Argentina; ^2^Institute of Cardiovascular Pathophysiology, School of Medicine, University of Buenos Aires, UBA-CONICET, Buenos Aires, Argentina

**Keywords:** metabolic syndrome X, neuroprotection, hypoxia, brain, oxidative stress, antioxidants

## Abstract

**Introduction:** Over the years the prevalence of metabolic syndrome (MetS) has drastically increased in developing countries as a major byproduct of industrialization. Many factors, such as the consumption of high-calorie diets and a sedentary lifestyle, bolster the spread of this disorder. Undoubtedly, the massive and still increasing incidence of MetS places this epidemic as an important public health issue. Hereon we revisit another outlook of MetS beyond its classical association with cardiovascular disease (CVD) and Diabetes Mellitus Type 2 (DM2), for MetS also poses a risk factor for the nervous tissue and threatens neuronal function. First, we revise a few essential concepts of MetS pathophysiology. Second, we explore some neuroprotective approaches in MetS pertaining brain hypoxia. The articles chosen for this review range from the years 1989 until 2017; the selection criteria was based on those providing data and exploratory information on MetS as well as those that studied innovative therapeutic approaches.

**Pathophysiology:** The characteristically impaired metabolic pathways of MetS lead to hyperglycemia, insulin resistance (IR), inflammation, and hypoxia, all closely associated with an overall pro-oxidative status. Oxidative stress is well-known to cause the wreckage of cellular structures and tissue architecture. Alteration of the redox homeostasis and oxidative stress alter the macromolecular array of DNA, lipids, and proteins, in turn disrupting the biochemical pathways necessary for normal cell function.

**Neuroprotection:** Different neuroprotective strategies are discussed involving lifestyle changes, medication aimed to mitigate MetS cardinal symptoms, and treatments targeted toward reducing oxidative stress. It is well-known that the routine practice of physical exercise, aerobic activity in particular, and a complete and well-balanced nutrition are key factors to prevent MetS. Nevertheless, pharmacological control of MetS as a whole and pertaining hypertension, dyslipidemia, and endothelial injury contribute to neuronal health improvement.

**Conclusion:** The development of MetS has risen as a risk factor for neurological disorders. The therapeutic strategies include multidisciplinary approaches directed to address different pathological pathways all in concert.

## Introduction

### Definition

Metabolic syndrome (MetS) is a disorder characterized by a cluster of conditions that increases the risk of developing cardiovascular disease (CVD) and Type 2 Diabetes Mellitus (DM2) (International Diabetes Federation, 2006). Currently, there are still controversies among the different Health Organizations on the selection criteria for this syndrome. The most accepted diagnostic tool is the global consensus described by the International Diabetes Federation (IDF) (International Diabetes Federation, 2006) that entails the presence of:

- Central obesity (based on waist circumference): ≥80 cm for women and ≥90 cm for men from the Hispanic background (values vary with ethnicity).
- Plus two or more of the following parameters:

1. **Hypertriglyceridemia**: ≥ 150 mg/dL, or under treatment for this lipid abnormality.
2. **HDL-cholesterolemia below recommendations**: <40 mg/dL in men and <50 mg/dL in women, or history of specific treatment for this lipid abnormality.
3. **Hypertension**: SBP ≥ 130 mmHg and/or DBP ≥ 85 mmHg over 24 h.
4. **Fasting Hyperglycemia**: ≥100 mg/dL, hyperinsulinemia, or DM2.

Metabolic syndrome (MetS) has been associated with hepatic steatosis, respiratory illness, osteoarticular disease, and cancer (Siegel and Zhu, 2009). The main mechanism implicated in the pathogenesis of MetS is the resistance to insulin (IR), namely the insufficient response to physiological insulin levels (Eckel, 2005). Other aspects that can shape MetS pathology are environmental stressors, mostly chemical, infections, lifestyle (sedentary habits, smoking, and nutritional factors), genetic predisposition, and other chronic diseases. These characteristics have one common denominator which is the generalized pro-oxidative status, in turn favoring free radical generation and resulting in oxidative stress (Roberts and Sindhu, 2009). The specific metabolic pathways typically affected by the development of MetS are discussed in more detail later in this work.

### Epidemiology

Metabolic syndrome (MetS) affects nearly 30% of the world population, associated with a 2–3-fold increase in morbidity and mortality compared with healthy people (Engin, 2017). In regard to the global statistics of MetS, an important item to highlight is the lack of unanimity on the diagnosis of this syndrome, specifically derived from the regional variation of the cut-off values for waist circumference linked to ethnicity (Borch-Johnsen, 2013). Therefore, the percentage calculated on a worldwide scale is only approximated and should be adjusted based on the prevalent ethnicity in a national scope.

Despite any criteria discrepancy, the need to reduce the prevalence of this disorder on a global scale becomes relevant. Not only does the presence of MetS reduce life expectancy and quality of life, but it also causes a financial burden derived from high health costs (Rask-Madsen and Kahn, 2012). For this reason, the World Health Organization (WHO) has as a priority to lower the worldwide prevalence of non-communicable diseases like CVD, DM2, and cancer, globally accounting for 63% of overall deaths. One example of this intended approach is the taxing of sweetened beverages.^1^

As aforementioned, MetS increases mortality and morbidity and is associated with accelerated aging (Rask-Madsen and Kahn, 2012). The aging process *per se* increases an individual's susceptibility to developing CVD or DM2. Interestingly, some reports substantiate how MetS even in the absence of CVD or DM2 also renders higher morbimortality (Borch-Johnsen, 2013).

The relevance of MetS in the modern industrialized society is undeniable. Its staggering global prevalence and concomitantly diminished quality of life rank this disorder as a major public health concern.

### Effects of metabolic syndrome on the nervous system

Over the years, the importance of MetS pertaining cardiovascular risk and progression to DM2 has been carefully studied and extensively divulged, for CVD is the leading cause of death worldwide.^2^ However, research has been scarce with regard to the effects of MetS on nervous tissue. In the recent years, the ever-growing evidence suggests a correlation between Alzheimer's disease (AD) and other cognitive impairments, and MetS. These results suggest that this syndrome does not only act as a risk factor for CVD and DM2 but also contributes to the progression toward AD (Kim and Feldman, 2015).

The nervous tissue has two vastly different cell populations: neuronal and glial cells. Neurons are highly specialized cells that propagate electrical stimulus in order to accomplish synaptic transmission, while the glia (composed mainly by astrocytes, oligodendrocytes, and microglia) is responsible for maintaining the homeostasis in nervous tissue. The brain depends upon glucose as its main source of energy, and a tight regulation of glucose metabolism and ATP reserves are critical for brain physiology (Mergenthaler et al., 2013; Brusco et al., 2014).

The aim of this review is:

1. First, to revise the pathophysiology of MetS and the consequences of the intrinsically altered metabolism in the nervous tissue.
2. To propose and explore different therapeutic approaches aimed at reducing the compromised neuronal function and neurodegenerative damage in MetS.

## Pathophysiology

### Overview

It is imperative to acknowledge that MetS develops in susceptible individuals bearing genetic factors and engaging in certain epigenetic unhealthy habits like a sedentary lifestyle, excessive consumption of high energy foods and drinks, smoking, and many others. This complex disorder is characterized by a sustained positive energy balance, which progressively breeds a mild inflammatory environment due to the activation of abnormal metabolic pathways (Kaur, 2014). Pivotal mechanisms implied in MetS were described in this review: hyperglycemia, insulin resistance (IR), inflammation and oxidative stress.

Patients with long-term MetS may be prone to develop diabetic encephalopathy due to the diabetogenic milieu, entailing moderate cognitive deficits, and both neurophysiological and structural changes in the brain (Biessels et al., 2002). Passos et al. demonstrated that the senescent cells had higher reactive oxygen species (ROS) concentration, dysfunctional mitochondria, more DNA double-strand breaks and shorter telomeres. It was also shown that mitochondrial ROS enhanced telomere-dependent senescence (Passos et al., 2007). Likewise, some authors showed the link between telomere length and metabolic disease suggesting increased cellular turnover and therefore accelerated cell aging (Bonomini et al., 2011; Kong et al., 2013). The increasing abdominal adiposity typical of the MetS is accompanied by accelerated telomere attrition (Révész et al., 2015).

### Hyperglycemia

It is well-known that a high concentration of blood glucose triggers diverse metabolic pathways. The most evident is the tricarboxylic acid (TCA) cycle, due to an abundant amount of substrate that in turn feeds the succeeding electron transport chain (ETC). This energy surplus creates an imbalance of partially reduced oxygen species favoring oxidant over antioxidant compounds and resulting in oxidative stress (Kawahito et al., 2009).

Another thoroughly described mechanism is the network of glycation reactions bringing forth oxidative stress alongside glucose toxicity (Kaneto et al., 1996). Advanced glycation end-products (AGEs) are formed as a result of these non-enzymatic reactions and promote inflammation by interacting with the receptor of AGEs (RAGE) in cells of the immune system (Gkogkolou and Böhm, 2012). The activation of the RAGE triggers abundant intracellular signaling pathways including kinases (e.g., MAP kinases, PI3 kinase), transcription factors as the nuclear factor-κB (NFκB), and the activator protein-1. This signaling cascade activates the further expression of cytokines, chemokines, enzymes, and growth factors resulting in an overall proinflammatory environment which leads to oxidative stress (Medzhito and Horng, 2009).

Moreover, hyperglycemia reduces antioxidant levels and increases the production of free radicals. The enzymes superoxide dismutase (SOD) and catalase or glutathione peroxidase involved in the antioxidant defense are down-regulated in the diabetic brain (Suresh Kumar and Menon, 1993; Makar et al., 1995; Miranda et al., 2007; Alvarez-Nölting et al., 2012). The possible source of oxidative stress in brain injury, however, also includes auto-oxidation of glucose, lipid peroxidation, and decreased tissue concentrations of low molecular weight antioxidants like reduced glutathione (GSH) (Reagan et al., 2000; Grillo et al., 2003; Ulusu et al., 2003; Muriach et al., 2006). This alteration of reduced glutathione levels may be related to an increased activity of the polyol pathway as this leads to a depletion of the NADPH required for enzymatic reduction of the oxidized glutathione (Preet et al., 2005).

Overall, hyperglycemia creates a pro-oxidative environment in live tissue involving various mechanisms. This condition causes a detrimental effect on various cells and tissues, especially those more vulnerable due to an insufficient antioxidant defense like the pancreatic β-cells (Kaneto et al., 1999).

### Insulin resistance

Insulin is well-known for its role in the regulation of glucose metabolism in the body. However, insulin has other roles in the central nervous system (CNS) pertaining cognitive processes, memory and synaptic plasticity (Zhao and Alkon, 2001). Accordingly, either the deficiency of insulin or hyperinsulinemia characteristic of type 1 or type 2 diabetes mellitus respectively could be associated with degenerative events in the brain (Xu et al., 2004).

Recent studies have suggested that the nervous system is also capable of developing resistance to insulin. This is possible even though neurons are not dependent on insulin, they bear insulin receptors and are insulin-responsive (Belfiore et al., 2009). Insulin receptors are expressed in brain areas as the olfactory bulb, cerebral cortex, hippocampus, hypothalamus and amygdala. Resistance to insulin in sensory neurons affects the cellular response to growth factors, leading to neurodegeneration, and neuropathy over time. In regard to mitochondrial metabolism, insulin regulates the PI3K/Akt (phosphatidylinositol 3-kinase/serine/threonine-specific Protein Kinase B) intracellular transduction signaling pathway (Stiles, 2009; Cheng et al., 2010) which promotes survival and growth in response to extracellular signals. This, in turn, affects neuronal mitochondria, resulting in increased oxidative stress (Fisher-Wellman and Neufer, 2012).

In addition, evidence has convincingly reported the involvement of the mechanistic mammalian target of rapamycin (mTOR) in cellular senescence. This pathway is activated by an insulin dependent signal. The mTOR modulates cell growth and cellular metabolism in response to growth factors, nutrients, and cellular energy conditions. The loss of mTOR signaling disrupts multiple responses in glucose metabolism, energy production, mitochondrial function, and cell growth (Blagosklonny, 2013).

### Inflammation

Inflammation is a biologically essential process which stands as a common denominator in various pathological circumstances. The inflammatory reaction is triggered in response to tissue damage in an attempt to restore tissue homeostasis via repairing mechanisms. In physiological conditions, the inflammatory reaction is controlled and self-limited. However, when the fine orchestrated regulation of these mechanisms is disrupted, the uncontrolled inflammatory response usually ends in collateral damage (Goldszmid and Trinchieri, 2012).

The amazingly complex immune system mediates the response to unknown stimuli. When the inflammatory response is unable to restore homeostasis, systemic, and cellular stress persists and physiological abnormalities develop (Okin and Medzhitov, 2012). Diabetes is an example of a chronic inflammatory disease (Pacher et al., 2007).

The production of ROS is a typical response to the stimulation of immune system cells (Meier et al., 1989, 1990). Both, acute and chronic inflammatory states have redox equilibrium alterations due to the increased generation of oxidizing agents (Pacher et al., 2007; Roberts et al., 2010; Li et al., 2013; Rochette et al., 2013). Toll-like receptors (TLRs) activate NFκB, a regulator of inflammation that is controlled by hundreds of genes and is also a redox-sensitive nuclear factor, are at a key juncture between the regulation of oxidative stress and inflammation.

Different experimental models from studies have described that the activation of NFκB and proinflammatory cytokines is associated to neuronal dysfunction, neuronal loss and impaired cognitive function (Mattson and Camandola, 2001; Vincent et al., 2002; Cai and Liu, 2012; Li et al., 2013). Activated NFκB can lead to oxidative stress-induced cell dysfunction or death due to the induction of cytotoxic products, which exacerbate inflammation and promote apoptosis (Pahl, 1999; Morgan and Liu, 2011).

Overnutrition is considered as an independent environmental factor which activates the innate immune system and triggers an atypical form of inflammation leading to metabolic dysfunction in the CNS (Cai and Liu, 2012). This inflammatory cascade can also affect the hypothalamus and impair appetite control, energy expenditure, carbohydrate and lipid metabolism, and blood pressure homeostasis (Kahn and Flier, 2000; Lam et al., 2005; Meister, 2007; Schenk et al., 2008; Zhang et al., 2008; Shoelson and Goldfine, 2009). The molecular pathway involved in this dysfunction is the activation of IKKβ/NFκB (Sonoda et al., 2008; Cai, 2009; Lumeng and Saltiel, 2011).

The endoplasmic reticulum (ER) also plays a key role in the metabolic imbalance caused by oxidative stress since it depends on IKKβ/NFκB pathway activity (Zhang et al., 2008; Purkayastha et al., 2011) and causes ROS accumulation (Cullinan and Diehl, 2006). Sustained exposure to high levels of blood glucose promotes oxidative stress generating oxidative free radicals and aberrant protein folding (Cullinan and Diehl, 2006).

Zhang et al. have reported an increase in the expression of C/EBP (CCAAT/enhancer-binding protein) homologous protein (CHOP) in the hippocampus of diabetic rats. This increase suggests that CHOP-ER stress-mediated apoptosis could be caused by hyperglycemia, impairing hippocampal synapses, and promoting diabetic cognitive dysfunction (Zhang et al., 2013).

### Oxidative stress

**Figure 1:**
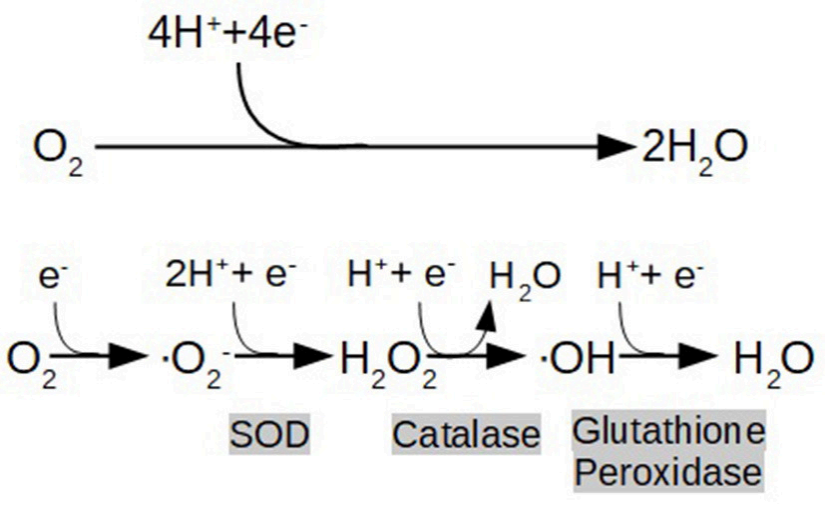
End metabolic pathway of electron transport chain (ETC), depicting the reduction of oxygen (O_2_) to water (H_2_O), and the formation of reactive oxygen species (ROS) as a consequence of its partial reduction: O_2_ (Superoxide) H_2_O_2_ (hydrogen peroxide), OH (hydroxyl radical). Highlighted in gray are shown the enzymes that catalyze the reactions (SOD:Superoxide Dimutase).

As previously described, IR, inflammation, and hyperglycemia all induce oxidative stress. The current concept of oxidative stress conceived by Helmut and Jones is an “imbalance between oxidants and antioxidants in favor of the oxidants, leading to a disruption of redox signaling and control, and/or molecular damage” (Sies and Jones, 2007). Free radicals are highly unstable and reactive molecules which disrupt protein structure and modify the physicochemical properties of membranes resulting in organelles and cell damage. Some cardinal examples of free radicals are the well-known ROS, which are byproducts of the partial reduction of O_2_ (Figure 1). This fundamental reaction occurs in Complex IV of the ETC during physiological respiration (Mitchell, 2012).

It is known that the appearance of oxygen in our world's atmosphere is directly correlated with the origin of the powerhouse organelles in cells: the mitochondria (Poderoso, 2016). The mitochondria not only supply the cell with a greater amount of ATP compared with other metabolic pathways but also play important roles in signaling apoptosis, thermoregulation, and other vital processes. The adaptation to an aerobic lifestyle benefited eukaryotic cells in many ways but also brought complications. Unfortunately, the aerobic environment generates ROS (Mitchell, 2012), and mitochondria are the main cellular source of ROS. Metabolically active tissues as the liver, heart, and brain are the major contributors of ROS to the body (Boveris and Repetto, 2016).

The amount of ROS, normally produced in low concentrations as byproducts of physiological processes like respiration, is regulated by a variety of enzymes and other molecules with antioxidant properties (Glutathione Peroxidase, Glutathione Reductase, SOD, Vitamin C, Vitamin E, and the β-carotenes) (Birben et al., 2012). When the redox homeostasis is affected, ROS are overproduced exceeding the physiological antioxidant capacity resulting in oxidative stress and causing structural and conformational changes in mitochondrial proteins, lipids, and nuclear material, thereby impairing their function. Mitochondrial dysfunction in particular, eventually leads to nerve cell damage. Since the nervous tissue is highly metabolically active and critically dependent on energy supply, it is unable to work properly in ATP shortcoming conditions (Bhat et al., 2015).

In diabetic patients, aging may be associated with brain dysfunction supported by the link between aging with cell death and oxidative stress mediated by free radicals (Beckman and Ames, 1998). In these pathological conditions, cellular stress triggers oxidative mitochondrial damage, which in turn may lead to apoptosis and/or necrosis (Merad-Boudia et al., 1998). Apoptosis-induced oxidative stress has been linked to neurogenesis inhibition (Cui et al., 2006). Alteration in mitochondrial the ETC, ROS formation, dysfunction of mitochondrial metabolism, and oxidative stress are recognized as the main protagonists in complications related to diabetes (Moreira et al., 2009).

Likewise, many studies have revealed that homocysteine, associated with endothelial dysfunction, is responsible for the release of hydrogen peroxide which causes cellular oxidative stress and inflammation mediated by cell injury *in vitro* (Rozycka et al., 2013). There is consensus that the activated microglia is an important key mediator of proinflammatory and neurotoxic factors involved in the progression of PD and AD. These proinflammatory and neurotoxic factors include cytokines like interleukin-6 (IL-6) and interleukin-1β (IL-1β), the tumor necrosis factor-α (TNF-α) and ROS, consequently contributing to oxidative stress (Bayarsaikhan et al., 2015).

Inexorably, altered metabolic homeostasis affecting multiple metabolic pathways in MetS becomes the driving force toward oxidative stress. The MetS should be regarded not only as a predictor of CVD and DM2 but also as a silent threat to cognitive performance and a risk for neurodegeneration (Kim and Feldman, 2015). Timely assessment of risk for MetS and its related conditions may be recommended.

### Hypoxia

Metabolic syndrome (MetS) is associated with an increased risk of cerebrovascular diseases, including cerebral ischemia (Aoqui et al., 2014). The abnormal metabolic pathways involved in MetS affect a broad variety of tissues, organs and systems, including the cardiovascular system. Microvascular dysfunction is particularly associated with MetS (Czernichow et al., 2010). This is not surprising since DM2 progresses toward micro and macrovascular dysfunction (Mitchell, 2012). Furthermore, high blood pressure, increased pulse wave velocity suggesting increased arterial stiffness, and low capillary density were found in individuals with MetS (Greenstein et al., 2009). Vascular degeneration brings forth a flawed circulation, which in turn leads to hypoxia in target organs like the brain.

There is ever-growing evidence for obesity associated with changes in perivascular adipose tissue, which gives forth an altered vasoactive tone of the microvasculature (Obadia et al., 2017). Some key factors that may play a crucial role regarding these vascular modifications are cardinal molecules that are increased substantially in MetS: free fatty acids and adipokines (TNF-α) (Greenstein et al., 2009). Additionally, not only do individuals with MetS have a higher likelihood of microvascular dysfunction, but they are also more susceptible to damage during ischemia-reperfusion events (Aoqui et al., 2014).

The cluster of these elements poses important risk factors toward producing hypoxia in tissues that have a strict oxygen supply requirement, such as nervous tissue. The neurovascular unit (composed of neurons, astrocytes and endothelial cells) is the structure in charge of maintaining the brain's homeostasis (Gorelick et al., 2011). Studies show that the dysfunction of this unit plays a crucial role regarding the onset of neurodegenerative conditions, such as AD (Zlokovic, 2008; Grammas, 2011; Marchesi, 2014). In fact, reports have associated stroke patients with progression toward AD, where hypoxic/ischemic injury promotes the formation of β-amyloid plaque (Guglielmotto et al., 2009).

## Neuroprotection

Neuroprotection encompasses the therapeutic actions to prevent or limit the progression of neuronal degeneration (Orsini et al., 2016). A vast array of noxious stimuli can trigger damage in nervous tissue. Among these, altered metabolic pathways and hypoxia are the main focus of this review (Marcano Torres, 2004).

The therapeutic approaches regarding neuroprotection discussed in this review comprise three instances: lifestyle habits, MetS symptoms medication (hypertension, hyperglycemia, and dyslipidemia), and antioxidant treatment.

### Lifestyle: nutritional habits and exercise

A fundamental cornerstone of neuroprotection in the context of MetS concerns striving toward physical wellness obtained by a balanced diet and exercise through a multidisciplinary approach. This item alone can revert hypertension, hyperglycemia, and dyslipidemia, without the need to implement medication (Pitsavos et al., 2006).

#### Nutritional habits

Reducing daily calorie intake and adopting a dietary style like the Mediterranean diet or the Dietary Approaches to Stop Hypertension (DASH) is advisable. While both dietary strategies improve the patient biochemical profile, the DASH was more beneficial in normalizing blood pressure (Kaur, 2014). The Adult Treatment Panel III (ATP III) recommends a diet for cholesterol management containing 25–35% of total fat to reduce the low density-cholesterol (LDL-C) fraction level (National Cholesterol Education Program, 2001). We have reported that chronic consumption of cola beverages impairs metabolic homeostasis increasing glycemia, cholesterolemia, triglyceridemia, and systolic blood pressure. Systolic blood pressure and most of the biochemical parameters normalize after switching cola beverages to tap water over a sustained washout period. However, hypertriglyceridemia is resistant and persists long after discontinuing cola consumption (Milei et al., 2011; Otero-Losada et al., 2014, 2015, 2016a).

#### Exercise

Health professionals should indicate exercise programs, such as 30 min or more of moderate-intensity physical activity and preferably all days of the week (Thompson et al., 2003). Continuing evidence from our research reports a beneficial effect of exercise on pancreatic morphology in long-term cola-drinking rats. These results support the by now accepted positive correlation between exercise and physical wellness (Otero-Losada et al., 2016b).

However, when modifying lifestyle habits does not revert pathological values, it is time to take the next level of action including a pharmacological therapeutic approach. Nevertheless, physical wellness should always compliment the medication.

### Medication targeted toward mets cardinal symptoms

The cardinal symptoms of MetS with indicated pharmacological treatment are hypertension, dyslipidemia, and hyperglycemia.

#### Hypertension

At present, a vast array of antihypertensive drugs is available (Gupta and Guptha, 2010). However, the election of a specific pharmacological association should consider a holistic view of each patient, always bearing in mind the individual idiosyncrasy and any other relevant factor (Gutiérrez, 2001). There is a certain structure regarding the course of drug administration that is validated by clinical trials in hypertensive patients. Treatment should initiate with a mono-drug treatment and only escalate indicating additional drugs with different mechanism of action in patients that do not normalize their blood pressure (Rivas-Chávez et al., 2007).

The first line drugs in pharmacological therapy^3^:

- Angiotensin Converting Enzyme (ACE) inhibitors/Angiotensin II Receptor Blockers (ARBs) which inhibit receptor binding to angiotensin II.
- Dihydropyridine Calcium Channel Blockers (CCBs) which inhibit Ca^2+^ influx into smooth muscle cells in vessels.
- Thiazide Diuretics which inhibit the reabsorption of Na^+^ and Cl^−^ in the distal tubules of the nephron.

The second line drugs in pharmacological therapy are administered to patients with risk factors or developing side effects from the first-line drugs (Gutiérrez, 2001; Sweitzer, 2003; De Luis Román et al., 2008).

#### Dyslipidemia

The pharmacological approach to normalize blood lipids comprises a variety of drugs indicated according to the patient's specific abnormality (Tonkin and Byrnes, 2014). In patients with MetS, the pharmacological goal is to decrease the LDL-C fraction and triglycerides, and increase the HDL-C fraction in blood. The classical first-line drugs involve statins, fibrates and inhibitors of cholesterol absorption while thiazolidinediones, GLP-1 agonists, and DPP-4 inhibitors seem to be promising therapeutic alternatives (Binesh Marvasti and Adeli, 2010).

Statins decrease LDL-C level acting as inhibitors of the 3-hydroxy-3-methylglutaryl coenzyme A (HMG-CoA) reductase, which is the key enzyme in cholesterol endogenous synthesis (Baigent et al., 2010). Most frequent adverse side effects of long-term treatment with statins are hepatotoxicity and myopathy (Pescio, 2001).

Ezetimibe, the standard cholesterol absorption inhibitor, impairs cholesterol luminal transport in the small intestine by the enterocyte (Tonkin and Byrnes, 2014). This drug lowers blood level of LDL-C and its effect increases when associated with a statin (Binesh Marvasti and Adeli, 2010).

Another drugs used in MetS are the fibrates, which activate peroxisome proliferator-activated receptors (PPARs) eventually improving lipid and carbohydrate metabolism. The fibrates are very effective at lowering blood triglycerides, reducing LDL-C, and increasing HDL-C as well (Staels et al., 1998). The thiazolidinediones are insulin-sensitizing drugs (i.e., rosiglitazone) that also act as PPARs stimulators and are used to normalize glycemia (Krentz et al., 2008).

The incretins, typically, the glucagon-like peptide-1 (GLP-1) and the glucose-dependent insulinotropic peptide (GIP), are gut hormones secreted by the enteroendocrine cells within minutes after ingestion and they stimulate insulin secretion after eating (Ahrén, 2003). Their use is promising in patients with DM2 (Meier and Nauck, 2006). The dipeptidyl peptidase-4 (DPP-4) is the enzyme responsible for lowering the level of incretin in blood to the basal level. Hence, the administration of DPP-4 inhibitors increases insulinemia (Kendall et al., 2006).

#### Hyperglycemia

The leading non-insulin antidiabetic pharmacological groups are the insulin-sensitizing drugs (biguanides and thiazolidinediones), insulin secretagogues (sulfonylureas, meglitinides, and incretins), and starch blockers (α-glucosidase inhibitors) (Rockville, 2007; Zárate et al., 2010; Powers and D'Alessio, 2012). The preferred drugs in MetS are those that do not have a direct effect on pancreatic β-cells, such as biguanides (metformin), thiazolidinediones (rosiglitazone), and α-glucosidase inhibitors (acarbose) (Mamedov and Shishkova, 2007). The main advantage of these euglycemic agents is avoiding hypoglycemia as a side effect (Vlckova et al., 2009).

Data describing clinical evidence concerning anti-MetS treatment and its effect on cognitive impairment is summarized in Table 1.

**Table 1:** Effect of anti-MetS treatment on cognitive processes, and brain circulation and metabolism in clinical trials.

**Symptoms**	**Drug**	***n***	**Sample Characteristics**	**Study Time**	**Results**	**References**
HYPERTENSION	ACE inhibitor	616	Mild/Moderate AD	4-year prospective	Mini-Mental State Examination (MMSE) decline between control and placebo	Soto et al., 2013
	CCB	18,423	Elderly hypertensive patients older than 60 years	12-year prospective	CCB use significantly reduced the risk of total dementia, Alzheimer's dementia, and vascular dementia	Hwang et al., 2016
	Thiazide Diuretics	3,417	Elderly hypertensive population with AD	7-year prospective	Use of any anti-hypertensive medication was associated with lower incidence of AD. Thiazides were associated with the greatest reduction of AD risk	Chuang et al., 2014
HYPERGLYCEMIA	Metformin (Biguanides)	20	Mild cognitive impairment or mild dementia due to AD	16-week prospective	Metformin was associated with improved executive functioning, and trends suggested improvement in learning/memory and attention	Koenig et al., 2017
	Pioglitazone (TZD)	42	Mild AD with type II Diabetes Mellitus	6-month prospective	The pioglitazone group improved cognition and regional cerebral blood flow in the parietal lobe	Sato et al., 2011
	Rosiglitazone (TZD)	30	Mild AD or amnestic mild cognitive impairment	6-month prospective	Relative to the placebo, better delayed recall, and selective attention	Watson et al., 2005
	GLP-1 agonists	9	Healthy, Caucasian males with a mean age of 22 years old	4-week prospective	Ensures less fluctuation of brain glucose levels	Gejl et al., 2012
DYSLIPIDEMIA	Simvastatin	46	45–64 year old participants with normal cognition, grouped by high or normal cholesterol	12-month prospective	Reduction of p-tau in CSF seen in patients who had high initial baseline LDL cholesterol	Li et al., 2017

*ACE, angiotensin converting enzyme; CCB, calcium channel blockers; TZD, thiazolidinedione; CSF, cerebral-spinal fluid; LDL, low-density lipoprotein*.

### Medication targeting oxidative stress

As described in this review, the common denominator of various pathological pathways is an overall pro-oxidative status. The use of diverse antioxidative strategies has been evaluated to mitigate oxidative stress, mainly in diabetic neuropathy (Spychalowicz et al., 2012).

#### Antioxidant drugs

Among the wide variety of antioxidant compounds, the following have been thoroughly studied:

- Vitamins A, C, and E
- Flavonoids
- Alpha-lipoic acid (ALA)
- Lutein and docosahexaenoic acid (DHA)

Other antioxidants have shown neuroprotection as well. Such is the case of the amino acids Taurine, acetyl L-carnitine, N-acetylcysteine (Negre-Salvayre et al., 2008; Shakher and Stevens, 2011; Wang et al., 2011; Hosseini and Abdollahi, 2013), synthetic ROS scavengers like tempol and SOD mimetics (De Silva and Miller, 2016), and resveratrol which decreases the activity of NF-κβ. Also, TNF-α, IL-6, and Cox-2, angiopoietin-1 which protects against brain stroke (Nguyen et al., 2012), the anti-inflammatory cytokines IL-10 and IL-8 (Shukla et al., 2017), mitochondrial antioxidants as the coenzyme Q10 (Chew and Watts, 2004), and uncoupler proteins (UCPs) (Harper et al., 2001; Green et al., 2004) have neuroprotective effects. Some of these drugs as tempol and the anti-inflammatory cytokines have improved the response to hypoxic events reducing the spread of microhemorrhages (Han et al., 2015; Shukla et al., 2017).

Vitamins A, C, and E are dietary antioxidants capable of directly reducing free radicals and participating in the recycling of antioxidant cofactors (Maritim et al., 2003). The antioxidant vitamins also boost the immune system, preserve DNA structure avoiding oxidative damage, and alleviate diabetic neuropathy symptomatology, all linked with oxidative stress reduction (Niedowicz and Daleke, 2005; Rahman et al., 2006; Valko et al., 2007; Salah et al., 2010).

Several subclasses of flavonoids are free radical scavengers found in plants (Nijveldt et al., 2001; Arts and Hollman, 2005; Nettleton et al., 2006; Lukačínová et al., 2008). Some examples are: proanthocyanidin, luteolin, hesperidin, fisetin, epigallocatechin-gallate, rutin, and quercetin have been shown to possess antioxidant activities which protect against diabetic neuropathy (Al-Enazi, 2003; Cui et al., 2008; Ibrahim, 2008; Maher et al., 2011; Wang et al., 2011; Baluchnejadmojarad and Roghani, 2012; Ferreira et al., 2013).

Alpha-lipoic acid (ALA), an amphiphile antioxidant molecule, has proved to be likely the most successful antioxidant in clinical trials (Vallianou et al., 2009). Both ALA and its active metabolite (DHLA), act at different levels as antioxidants; they are free radical scavengers, inhibit the hexosamine and AGEs pathways, and are also involved in the intracellular regeneration of ascorbic acid (vitamin C), alpha-tocopherol (vitamin E), and oxidized glutathione (GSSG) (Packer et al., 2001; Du et al., 2008; Vallianou et al., 2009). Many experimental models support the beneficial effects of ALA in diabetic neuropathy (Nagamatsu et al., 1995; Evans et al., 2002; Ametov et al., 2003; Baydas et al., 2004; Ziegler et al., 2004, 2006, 2011; Tankova et al., 2005; Du et al., 2008; Huang and Gitelman, 2008; Gianturco et al., 2009; Vallianou et al., 2009).

The advantageous treatment with lutein and DHA in the brain of diabetic animals, and the way that these substances were able to mitigate the intrinsically oxidative environment in diabetes has also been studied (Muriach et al., 2006; Arnal et al., 2010). Moreover, treatment with DHA improved memory and learning skills in patients with Alzheimer's disease, related to a decrease in the concentration of lipid peroxide and ROS (Hashimoto et al., 2005).

#### Mitigating oxidative stress pathways

Diverse research projects have studied the downstream sequence of reactions and biochemical avenues undergoing oxidative stress as possible targets for the treatment of oxidative neuropathy. For the purpose of this review, we will focus on Aldose Reductase Inhibitors (ARIs), PKC Inhibitors, and Anti-AGE Agents.

##### Aldose Reductase Inhibitors (ARIs)

The aldose reductase enzyme participates in the synthesis of sorbitol and fructose. The ARIs decrease the amount of glucose entered into the polyol pathway avoiding neuronal accumulation of sorbitol and fructose. Based on the positive effect of ARIs administration in neuropathies caused by oxidative stress (Hotta et al., 1996; Yagihashi et al., 2001), clinical trials were conducted to test the effects of Fidarestat (SNK-860) (Hotta et al., 2001), Epalrestat (Hotta et al., 2006; Ramirez and Borja, 2008; Sharma and Sharma, 2008), and Ranirestat (AS-3201) (Bril and Buchanan, 2004; Bril et al., 2009). To date, only Epalrestat has a license in Japan while the other two were removed from the market (Casellini and Vinik, 2006; Kawai et al., 2010; Schemmel et al., 2010).

##### Protein Kinase C (PKC) Inhibitors

The PKC enzyme participates in the activation of key regulatory proteins responsible for the synthesis of neurotransmitters, and it is essential for nerve impulse conduction. Different studies have shown that PKC participates in alleviating neuropathic pain (Chattopadhyay et al., 2008; Norcini et al., 2009). The specific PKC-1b inhibitor Ruboxistaurin improved axonal velocity and endoneurial blood flow in diabetic rats (Nakamura et al., 1999). In clinical trials, Ruboxistaurin slowed down the progression of diabetic neuropathy but was not effective in suppressing the neuropathic symptomatology (Vinik et al., 2005).

##### Anti-advanced glycation end products (AGEs) Agents

Some drugs may prevent or inhibit AGEs formation and accumulation. The anti-AGEs drug's family is also responsible for disrupting the interaction between AGEs and AGEs receptors (RAGEs), which would otherwise magnify oxidative damage. Some examples of these agents are Benfotiamine, Aminoguanidine, Aspirin, and Rapamycin (Haupt et al., 2005; Edwards et al., 2008).

- Benfotiamine increases the activity of the Transketolase enzyme which is responsible for directing AGE substrates to the phosphate pentose pathway, consequently reducing hyperglycemic damage. Benfotiamine also inhibits the increase in UDP-N-acetylglucosamine (UDP-GlcNAc), which in turn limits the substrate's capacity to enter the hexosamine pathway, reducing AGEs production (Stirban et al., 2007; Balakumar et al., 2010).
- Aminoguanidine reacts with 3-deoxyglucosone, an AGE precursor, inactivating the reactive carbonyl residues essential to enzymatic activity, and preventing AGEs formation. Studies on this compound, however, have been discontinued due to its toxicity (Yan et al., 2008).
- *In Vitro* studies have shown that aspirin inhibits the production of pentosidine (an AGE) by accepting free radicals and ionic chelants in the presence of collagen (Urios et al., 2007).
- Ding et al. studied the activity of Rapamycin, an inhibitor of the proapoptotic mTOR-p53-Bax pathway, showing that Rapamycin injection 4 h after causing an injury, improved functional recovery. Recovery consisted of a reduction in microglial and macrophage activation, a decreased rate of apoptosis, and an improved neurobehavioral function (Ding et al., 2015).

### Our research

As already mentioned, one of our research lines investigates the effects of chronic cola beverages consumption in murine models. Clear evidence of beneficial effects of a wash-out period (diet) and exercise have been reported (Milei et al., 2011; Otero-Losada et al., 2014, 2015, 2016a,b).

Another study conducted in our institution included elderly patients of both sexes that attended periodical routine check-ups, with the purpose of evaluating the effectiveness of short-term antioxidant supplementation. The outcome of our study confirmed that antioxidant supplementation improved plasma biochemistry as a result of changes in oxidative metabolism, typically in those patients having low basal endogenous antioxidants concentration in plasma. Based on these results, we recommended measuring the basal level of plasma antioxidants before starting any supplementation with antioxidants in elderly cardiovascular patients, a particularly vulnerable population with special precautions to be endorsed. Not only adverse effects were not reported during the course of our study, but subjective observations as “feeling more vital” or experiencing a state of “general well-being” were declared (Otero-Losada et al., 2013).

Using an experimental model, we assessed the therapeutic benefits of medical grade ozone, a mixture of 0.05–5% of pure O_3_ and 95–99.5% of pure O_2_ according to the pharmaceutical legislation. We reported promising effects of medical ozone auto-hemotransfusion on the cardiovascular system injury. Pretreatment with ozone auto-hemotransfusion decreased neointimal proliferation and induced reendothelialization following a metal stent insertion (Barone et al., 2016). The protective mechanism whereby ozone reduces restenosis appears to involve the ozone well-described oxidative preconditioning upregulating the antioxidant enzymes, and improving the antioxidant response to an eventual injury (Barone et al., 2016).

Cognitive impairment or skills were not assessed in these experimental models. However, the promising outcome encourages pursuing further study of cognitive abilities as well.

### Clinical relevance regarding neurodegenerative diseases

Among neurodegenerative disorders, AD has been extensively studied regarding cognitive impairment in the setting of MetS (Hashimoto et al., 2005; Moreira et al., 2009; Mayeux and Stern, 2012; Zhang et al., 2013; Jayaraman and Pike, 2014; Kleinridders et al., 2014; Kim and Feldman, 2015). This tendency is reasonable since there is genetic evidence showing the apolipoprotein E (ApoE) as a common gene which links dementia, MetS, and diabetes (Zhang et al., 2017). Moreover, it is also crucial to bear in mind the extent of hardships that come hand in hand with AD (i.e., long-term functional dependence of the affected patients, the cost of living, quality of life, etc.), making this disorder an important public health issue (Agüero-Torres et al., 1998; Geldmacher et al., 2006). One of the challenges for public health is to identify risk factors, and the MetS is definitely a prevailing one. The compelling association between altered metabolic states and dementia may provide insight on a therapeutic approach that can delay cognitive impairment, targeting the underlying pathophysiological pathways of MetS (de la Monte, 2017).

Staggering evidence demonstrates that AD pathogenesis is strongly associated with oxidative stress, inflammation, and insulin, glucose, and lipid dysregulation; all of these pathological pathways are present in MetS and other altered metabolic states (Rojas-Gutierrez et al., 2017). Consequently, a vast number of studies have shown anti-MetS treatments to improve diverse aspects of cognition in patients with AD (Chen et al., 2016; de la Monte, 2017; Rojas-Gutierrez et al., 2017). An example of said treatments include drugs like gut incretins, thiazolidinediones, and metformin, which show promising results regarding cognitive impairment in animal and clinical trials, improving the effects of insulin via different mechanisms (Chen et al., 2016). Table 1 summarizes some data observed in clinical trials pertinent to anti-MetS treatment. Furthermore, studies on anti-oxidative treatment in patients with AD also present supportive results. For instance, flavonoids scavenge free radicals and have shown to promote neuronal survival in the hippocampus (Venkatesan et al., 2015). Another promising antioxidant treatment involves the administration of the synthetic S-acyl derivative of thiamine (vitamin B1) Benfotiamine in clinical trials reporting a long-term cognitive improvement, suggesting a possible disease-modifying therapy (Pan et al., 2016). Also, both AD and IR led to deregulation of the mTOR pathway, and treatment with the mTOR inhibitor Rapamycin resulted in learning and memory improvement in AD (Vieira et al., 2017).

Ultimately, the emerging global epidemic of neurodegenerative disorders as the world population ages is of great concern (Brookmeyer et al., 2007). Any insight regarding neuroprotection and delaying the onset of cognitive impairments is of utmost value. The ever-growing evidence of the association between AD and MetS not only allows a deeper comprehension of the pathogenic circuits but also lays the foundations for developing new therapeutic approaches aimed at normalizing both shared and intertwined pathological pathways underlying the two conditions (Agüero-Torres et al., 1998; Geldmacher et al., 2006; Venkatesan et al., 2015; Chen et al., 2016; Pan et al., 2016; de la Monte, 2017; Rojas-Gutierrez et al., 2017; Vieira et al., 2017; Zhang et al., 2017).

## Conclusion

Metabolic Syndrome (MetS) is an ever-growing disorder affecting up to 25% of the population in industrialized countries increasing morbimortality. Given these proportions, it has become an epidemic of public health concern. Increasing evidence shows that MetS is a risk factor for neurological disorders, beyond its classical association with CVD and DM2.

Hyperglycemia, IR, inflammation, oxidative stress, and hypoxia are key pathological pathways associated with MetS. It is a question of time until this cluster of conditions results in tissue damage in target organs such as the brain and the microvasculature that irrigates the nervous system. Oxidative stress and hypoxia are actually linked to neurological diseases like Alzheimer's and Parkinson's (Kim and Feldman, 2015).

The therapeutic strategies suggested in this review entail multidisciplinary interventions involving different pathological pathways in concert. These include improving lifestyle and daily habits (diet and exercise), treating the cardinal symptoms of MetS, and reducing the pro-oxidative load in affected patients. Antioxidant therapy is not routinely used in MetS, although extensive research relative to its benefits in diabetic neuropathy is available. Hence, we consider that diminishing the pro-oxidative status in patients with MetS may play a critical role in reducing brain hypoxic damage and behavioral deficits.

## Author contributions

ME: Lead author; MN and BP (B.Sc.): Data collection; FB: Proofreading; JG: Laboratory research; JM: Editing; MO-L: Editing and proofreading. NL: Has interpreted the data, revised for intellectual content, approved the final version of the work to be published, and may account for a research work properly accomplished.

### Conflict of interest statement

The authors declare that the research was conducted in the absence of any commercial or financial relationships that could be construed as a potential conflict of interest.

## Footnotes

^1^WHO Mid-term Programmatic and Financial Report for 2016–2017 including audited financial statements for 2016 (WHA 70/40) (http://www.who.int/about/finances-accountability/reports/en/).

^2^World Health Organization. The top 10 causes of death (Fact sheet) Updated January 2017 http://www.who.int/mediacentre/factsheets/fs310/en/.

^3^National Institute for Health and Clinical Excellence Hypertension (CG127). http://www.nice.org.uk/guidance/cg127. Accessed October 10, 2017.

## References

[B1] Agüero-Torres H. Fratiglioni L. Guo Z. Viitanen M. von Strauss E. Winblad B. (1998). Dementia is the major cause of functional dependence in the elderly: 3-year follow-up data from a population-based study. Am. J. Public Health 88, 1452–1456. 10.2105/AJPH.88.10.1452 9772843 PMC1508485

[B2] Ahrén B. (2003). Gut peptides and type 2 diabetes mellitus treatment. Curr. Diab. Rep. 3, 365–372. 10.1007/s11892-003-0079-9 12975025

[B3] Al-Enazi M. M. (2003). Ameliorative potential of rutin on streptozotocin-induced neuropathic pain in rat. Afr. J. Pharm. Pharmacol. 7, 2743–2754. 10.5897/AJPP2012.1534

[B4] Alvarez-Nölting R. Arnal E. Barcia J. M. Miranda M. Romero F. J. (2012). Protection by DHA of early hippocampal changes in diabetes: possible role of CREB and NF-kB. Neurochem. Res. 37, 105–115. 10.1007/s11064-011-0588-x 21909958

[B5] Ametov A. S. Barinov A. Dyck P. J. Hermann R. Kozlova N. Litchy W. J.. (2003). The sensory symptoms of diabetic polyneuropathy are improved with α-lipoic acid: The Sydney trial. Diabetes Care 26, 770–776. 10.2337/diacare.26.3.770 12610036

[B6] Aoqui C. Chmielewski S. Scherer E. Eissler R. Sollinger D. Heid I.. (2014). Microvascular dysfunction in the course of metabolic syndrome induced by high-fat diet. Cardiovasc. Diabetol. 13:31. 10.1186/1475-2840-13-31 24490784 PMC3916304

[B7] Arnal E. Miranda M. Barcia J. Bosch-Morell F. Romero F. J. (2010). Lutein and docosahexaenoic acid prevent cortex lipid peroxidation in streptozotocin-induced diabetic rat cerebral cortex. Neuroscience 166, 271–278. 10.1016/j.neuroscience.2009.12.028 20036322

[B8] Arts I. C. Hollman P. C. (2005). Polyphenols and disease risk in epidemiologic studies. Am. J. Clin. Nutr. 81, 317S–325S. 10.1093/ajcn/81.1.317S 15640497

[B9] Baigent C. Blackwell L. Emberson J. Holland L. E. Reith C. Bhala N.. (2010). Efficacy and safety of more intensive lowering of LDL cholesterol: a meta-analysis of data from 170,000 participants in 26 randomised trials. Lancet 376, 1670–1681. 10.1016/S0140-6736(10)61350-5 21067804 PMC2988224

[B10] Balakumar P. Rohilla A. Krishan P. Solairaj P. Thangathirupathi A. (2010). The multifaceted therapeutic potential of benfotiamine. Pharmacol. Res. 61, 482–488. 10.1016/j.phrs.2010.02.008 20188835

[B11] Baluchnejadmojarad T. Roghani M. (2012). Chronic oral epigallocatechin-gallate alleviates streptozotocin-induced diabetic neuropathic hyperalgesia in rat: involvement of oxidative stress. Iran. J. Pharm. Res. 11, 1243–1253. 24250559 PMC3813147

[B12] Barone A. Otero-Losada M. Grangeat A. M. Cao G. Azzato F. Rodríguez A.. (2016). Ozonetherapy protects from in-stent coronary neointimal proliferation. Role of redoxins. Int. J. Cardiol. 223, 25–261. 10.1016/j.ijcard.2016.07.177 27541668

[B13] Bayarsaikhan E. Bayarsaikhan D. Lee J. Son M. Oh S. Moon J.. (2015). Microglial AGE-albumin is critical for neuronal death in Parkinson's disease: a possible implication for theranostics. Int. J. Nanomedicine 10, 281–292. 10.2147/IJN.S95077 27601894 PMC5003553

[B14] Baydas G. Donder E. Kiliboz M. Sonkay E. Tuzku M. Yasar A.. (2004). Neuroprotection by α-lipoic acid in streptozotocin-induced diabetes. Biochemistry 69, 1001–1005. 10.1023/B:BIRY.0000043542.39691.95 15521814

[B15] Beckman K. B. Ames B. N. (1998). The free radical theory of aging matures. Physiol. Rev. 78, 547–581. 10.1152/physrev.1998.78.2.547 9562038

[B16] Belfiore A. Frasca F. Pandini G. Sciacca L. Vigneri R. (2009). Insulin receptor isoforms and insulin receptor/insulin-like growth factor receptor hybrids in physiology and disease. Endocr. Rev. 30, 586–623. 10.1210/er.2008-0047 19752219

[B17] Bhat A. H. Dara K. B. Anees S. Zargar M. A. Masood A. Sofi M. A.. (2015). Oxidative stress, mitochondrial dysfunction and neurodegenerative diseases; a mechanistic insight. Biomed. Pharmacother. 74, 101–110. 10.1016/j.biopha.2015.07.025 26349970

[B18] Biessels G. J. van der Heide L. P. Kamal A. Bleys R. L. Gispen W. H. (2002). Ageing and diabetes: implications for brain function. Eur. J. Pharmacol. 441, 1–14. 10.1016/S0014-2999(02)01486-3 12007915

[B19] Binesh Marvasti T. Adeli K. H. (2010). Pharmacological management of metabolic syndrome and its lipid complications. Daru 18, 146–154. 22615610 PMC3304358

[B20] Birben E. Sahiner U. M. Sackesen C. Erzurum S. Kalayci O. (2012). Oxidative stress and antioxidant defense. World Allergy Organ. J. 5, 9–19. 10.1097/WOX.0b013e3182439613 23268465 PMC3488923

[B21] Blagosklonny M. V. (2013). TOR-centric view on insulin resistance and diabetic complications: perspective for endocrinologists and gerontologists. Cell Death Dis. 4:e964 10.1038/cddis.2013.506 24336084 PMC3877573

[B22] Bonomini F. Rodella L. F. Moghadasian M. Lonati C. Coleman R. Rezzani R. (2011). Role of apolipoprotein E in renal damage protection. Histochem. Cell Biol. 135, 571–579. 10.1007/s00418-011-0815-1 21573735

[B23] Borch-Johnsen K. (2013). Epidemiology of the Metabolic Syndrome. Vienna: Springer.

[B24] Boveris A. Repetto M. G. (2016). Mitochondria are the main cellular source of O_2_-, H_2_O_2_ and oxidative stress, in Biochemistry of Oxidative Stress, Physiopathology and Clinical Aspects, Vol. 16, eds Gelpi R. J. Boveris A. Poderoso J. J. (New York, NY: Springer International Publishing), 23–36.

[B25] Bril V. Buchanan R. A. (2004). Aldose reductase inhibition by AS-3201 in sural nerve from patients with diabetic sensorimotor polyneuropathy. Diabetes Care. 27, 2369–2375. 10.2337/diacare.27.10.2369 15451902

[B26] Bril V. Hirose T. Tomioka S. Buchananet R.. (2009). Ranirestat for the management of diabetic sensorimotor polyneuropathy. Diabetes Care 32, 1256–1260. 10.2337/dc08-2110 19366965 PMC2699746

[B27] Brookmeyer R. Johnson E. Ziegler-Graham K. Arrighi H. M. (2007). Forecasting the global burden of Alzheimer's disease. Alzheimers Dement. 3, 186–191. 10.1016/j.jalz.2007.04.381 19595937

[B28] Brusco H. A. López Costa J. J. Loidl C. F. (eds.). (2014). Tejido nervioso, in Histología Médico-Práctica, Vol. 1 (Amsterdam: Elsevier), 115–131.

[B29] Cai D. (2009). NF-κB-mediated metabolic inflammation in peripheral tissues versus central nervous system. Cell Cycle 8, 2542–2548. 10.4161/cc.8.16.9386 19633416

[B30] Cai D. Liu T. (2012). Inflammatory cause of metabolic syndrome via brain stress and NF- κB. Aging 2, 98–115. 10.18632/aging.100431 PMC3314172 22328600

[B31] Casellini C. M. Vinik A. I. (2006). Recent advances in the treatment of diabetic neuropathy. Curr. Opin. Endocrinol. Diabetes 13, 147–153. 10.1097/01.med.0000216963.51751.be

[B32] Chattopadhyay M. Mata M. Fink D. J. (2008). Continuous δ-opioid receptor activation reduces neuronal voltage-gated sodium channel (NaV1.7) levels through activation of protein kinase C in painful diabetic neuropathy. J. Neurosci. 28, 6652–6658. 10.1523/JNEUROSCI.5530-07.2008 18579738 PMC3321315

[B33] Chen Y. Zhang J. Zhang B. Gong C. X. (2016). Targeting insulin signaling for the treatment of Alzheimer's disease. Curr. Top. Med. Chem. 16, 485–492. 10.2174/1568026615666150813142423 26268336

[B34] Cheng Z. Tseng Y. White M. F. (2010). Insulin signaling meets mitochondria in metabolism. Trends Endocrinol. Metab. 21, 589–598. 10.1016/j.tem.2010.06.005 20638297 PMC3994704

[B35] Chew G. T. Watts G. F. (2004). Coenzyme Q10 and diabetic endotheliopathy: oxidative stress and the ‘recoupling hypothesis’. QJM 97, 537–548. 10.1093/qjmed/hch089 15256611

[B36] Chuang Y. F. Breitner J. C. S. Chiu Y. L. Khachaturian A. Hayden K. Corcoran C. (2014). Cache County Investigators. Use of diuretics is associated with reduced risk of Alzheimer's disease: the Cache County Study. Neurobiol. Aging 35, 2429–2435. 10.1016/j.neurobiolaging.2014.05.002 24910391 PMC4366136

[B37] Cui X. P. Li B. Y. Gao H. Q. Wei N. Wang W. L. Lu M. (2008). Effects of grape seed proanthocyanidin extracts on peripheral nerves in streptozocin-induced diabetic rats. J. Nutr. Sci. Vitaminol. 54, 321–328. 10.3177/jnsv.54.321 18797155

[B38] Cui X. Zuo P. Zhang Q. Li X. Hu Y. Long J.. (2006). Chronic systemic D-galactose exposure induces memory loss, neurodegeneration, and oxidative damage in mice: protective effects of R-alpha-lipoic acid. J. Neurosci. Res. 84, 647–654. 10.1002/jnr.20899 16710848

[B39] Cullinan S. B. Diehl J. A. (2006). Coordination of ER and oxidative stress signalling: the PERK/Nrf2 signalling pathway. Int. J. Biochem. Cell Biol. 38, 317–332. 10.1016/j.biocel.2005.09.018 16290097

[B40] Czernichow S. Greenfield J. R. Galan P. Jellouli F. Safar M. E. Blacher J.. (2010). Macrovascular and microvascular dysfunction in the metabolic syndrome. Hypertens Res. 33, 293–297. 10.1038/hr.2009.228 20075933

[B41] de la Monte S. M. (2017). Insulin resistance and neurodegeneration: progress towards the development of new therapeutics for Alzheimer's disease. Drugs 77, 47–65. 10.1007/s40265-016-0674-0 27988872 PMC5575843

[B42] De Luis Román D. Allerb R. Bustamante J. (2008). Aspectos terapéuticos de la dieta en la hipertensión arterial. NefroPlus 1, 39–46.

[B43] De Silva T. M. Miller A. A. (2016). Cerebral small Vessel disease: targeting oxidative stress as a novel therapeutic strategy? Front. Pharmacol 7:61. 10.3389/fphar.2016.00061 27014073 PMC4794483

[B44] Ding K. Wang H. Wu Y. Zhang L. Xu J. Li T.. (2015). Rapamycin protects against apoptotic neuronal death and improves neurologic function after traumatic brain injury in mice via modulation of the mTOR-p53-Bax axis. J. Surg. Res. 194, 239–247. 10.1016/j.jss.2014.09.026 25438952

[B45] Du X. Edelstein D. Brownlee M. (2008). Oral benfotiamine plus α-lipoic acid normalises complication-causing pathways in type 1 diabetes. Diabetologia 51, 1930–1932. 10.1007/s00125-008-1100-2 18663426

[B46] Eckel R. H. (2005). The metabolic syndrome. Lancet 365, 1415–1428. 10.1016/S0140-6736(05)66378-7 15836891

[B47] Edwards J. L. Vincent A. M. Cheng H. T. Feldman E. L. (2008). Diabetic neuropathy: mechanisms to management. Pharmacol. Therapeut. 120, 1–34. 10.1016/j.pharmthera.2008.05.005 18616962 PMC4007052

[B48] Engin A. (2017). The definition and prevalence of obesity and metabolic syndrome. Adv. Exp. Med. Biol. 960, 1–17. 10.1007/978-3-319-48382-5_1 28585193

[B49] Evans J. L. Heymann C. J. Goldfine I. D. AMDGavin L. A. (2002). Pharmacokinetics, tolerability, and fructosamine-lowering effect of a novel, controlled-release formulation of α-lipoic acid. Endoc. Pract. 8, 29–35. 10.4158/EP.8.1.29 11951812

[B50] Ferreira P. E. B. Lopes C. R. P. Alves A. M. P. Alves E. P. Linden D. R. Linden D. R.. (2013). Diabetic neuropathy: an evaluation of the use of quercetin in the cecum of rats. World J. Gastroenterol. 19, 6416–6426. 10.3748/wjg.v19.i38.6416 24151360 PMC3801312

[B51] Fisher-Wellman K. H. Neufer P. D. (2012). Linking mitochondrial bioenergetics to insulin resistance via redox biology. Trends Endocrinol. Metab. 23, 142–153. 10.1016/j.tem.2011.12.008 22305519 PMC3313496

[B52] Gejl M. Egefjord L. Lerche S. Vang K. Bibby B. M. Holst J. J.. (2012). Glucagon-like peptide-1 decreases intracerebral glucose content by activating hexokinase and changing glucose clearance during hyperglycemia. J. Cereb. Blood Flow Metab. 32, 2146–2152. 10.1038/jcbfm.2012.118 22929437 PMC3519409

[B53] Geldmacher D. S. Frolich L. Doody R. S. Erkinjuntti T. Vellas B. Jones R. W.. (2006). Realistic expectations for treatment success in Alzheimer's disease. J. Nutr. Health Aging 10, 417–429. 17066215

[B54] Gianturco V. Bellomo A. D'Ottavio E. Formosa V. Iori A. Mancinella M.. (2009). Impact of therapy with alpha-lipoic acid (ALA) on the oxidative stress in the controlled NIDDM: a possible preventive way against the organ dysfunction? Arch. Gerontol. Geriatr. 49, 129–133. 10.1016/j.archger.2009.09.022 19836626

[B55] Gkogkolou P. Böhm M. (2012). Advanced glycation end products: key players in skin aging? Dermato Endocrinol. 4, 259–270. 10.4161/derm.22028 23467327 PMC3583887

[B56] Goldszmid R. S. Trinchieri G. (2012). The price of immunity. Nat. Immunol. 13, 932–938. 10.1038/ni.2422 22990891

[B57] Gorelick P. B. Scuteri A. Black S. E. Decarli C. Greenberg S. M. Ladecola C.. (2011). Vascular contributions to cognitive impairment and dementia: a statement for healthcare professionals from the American Heart Association/American Stroke Association. Stroke 42, 2672–2713. 10.1161/STR.0b013e3182299496 21778438 PMC3778669

[B58] Grammas P. (2011). Neurovascular dysfunction, inflammation and endothelial activation: implications for the pathogenesis of Alzheimer's disease. J. Neuroinflammation 8:26. 10.1186/1742-2094-8-26 21439035 PMC3072921

[B59] Green K. Brand M. D. Murphy M. P. (2004). Prevention of mitochondrial oxidative damage as a therapeutic strategy in diabetes. Diabetes 53, S110–S118. 10.2337/diabetes.53.2007.S110 14749275

[B60] Greenstein A. S. Khavandi K. Withers S. B. Sonoyama K. Clancy O. Jeziorska M.. (2009). Local inflammation and hypoxia abolish the protective anticontractile properties of perivascular fat in obese patients. Circulation 119, 1661–1670. 10.1161/CIRCULATIONAHA.108.821181 19289637

[B61] Grillo C. A. Piroli G. G. Rosell D. R. Hoskin E. K. Mcewen B. S. Reagan L. P. (2003). Region-specific increases in oxidative stress and superoxide dismutase in the hippocampus of diabetic rats subjected to stress. Neuroscience 121, 133–140. 10.1016/S0306-4522(03)00343-9 12946706

[B62] Guglielmotto M. Aragno M. Autelli R. Gliberto L. Novo E. Colombatto S.. (2009). The up-regulation of BACE1 mediated by hypoxia and ischemic injury: role of oxidative stress and HIF1alpha. J. Neurochem. 108, 1045–1056. 10.1111/j.1471-4159.2008.05858.x 19196431

[B63] Gupta R. Guptha S. (2010). Strategies for initial management of hypertension. Indian J. Med. Res. 132, 531–542. 21150005 PMC3028941

[B64] Gutiérrez J. (2001). Tratamiento de la Hipertensión Arterial. Cambio de estilo de Vida.

[B65] Han B. H. Zhou M. L. Johnson A. W. Singh I. Liao F. Vellimana A. K.. (2015). Contribution of reactive oxygen species to cerebral amyloid angiopathy, vasomotor dysfunction, and microhemorrhage in aged Tg2576 mice. Proc. Natl. Acad. Sci. U.S.A. 112, E881–E890. 10.1073/pnas.1414930112 25675483 PMC4345564

[B66] Harper J. A. Dickinson K. Brand M. D. (2001). Mitochondrial uncoupling as a target for drug development for the treatment of obesity. Obesity Rev. 2, 255–265. 12119996 10.1046/j.1467-789x.2001.00043.x

[B67] Hashimoto M. Tanabe Y. Fujii Y. Kikuta T. Shibata H. Shido O. (2005). Chronic administration of docosahexaenoic acid ameliorates the impairment of spatial cognition learning ability in amyloid β-infused rats. J. Nutr. 135, 549–555. 10.1093/jn/135.3.549 15735092

[B68] Haupt E. Ledermann H. Köpcke W. (2005). Benfotiamine in the treatment of diabetic polyneuropathy—a three-week randomized, controlled pilot study (BEDIP study). Int. J. Clin. Pharmacol. Ther. 43, 71–77. 10.5414/CPP43071 15726875

[B69] Hosseini A. Abdollahi M. (2013). Diabetic neuropathy and oxidative stress: therapeutic perspectives. Oxidat. Med. Cell. Long. 2013:168039. 10.1155/2013/168039t 23738033 PMC3655656

[B70] Hotta N. Akanuma Y. Kawamori R. Matsuoka K. Oka Y. Shichiri M.. (2006). Long-term clinical effects of epalrestat, an aldose reductase inhibitor, on diabetic peripheral neuropathy: the 3-year, multicenter, comparative aldose reductase inhibitor-diabetes complications trial. Diabetes Care 29, 1538–1544. 10.2337/dc05-2370 16801576

[B71] Hotta N. Sakamoto N. Shigeta Y. Kikkawa R. Goto Y. (1996). Clinical investigation of epalrestat, aldose reductase inhibitor, on diabetic neuropathy in Japan: a multicenter study. J. Diabetes Complicat. 10, 168–172. 10.1016/1056-8727(96)00113-4 8807467

[B72] Hotta N. Toyota T. Matsuoka K. Shigeta Y. Kikkawa R. Kaneko T. (2001). SNK-860 diabetic neuropathy study group: clinical efficacy of fidarestat, a novel aldose reductase inhibitor, for diabetic peripheral neuropathy: a 52-week multicenter placebo-controlled double-blind parallel group study. Diabetes Care 24, 1776–1782. 10.2337/diacare.24.10.1776 11574441

[B73] Huang E. A. Gitelman S. E. (2008). The effect of oral alpha-lipoic acid on oxidative stress in adolescents with type 1 diabetes mellitus. Pediatr. Diabetes 9, 69–73. 10.1111/j.1399-5448.2007.00342.x 18221433

[B74] Hwang D. Kim S. Choi H. Oh I. H. Kim B. S. Choi H. R.. (2016). Calcium-channel blockers and dementia risk in older adults- National Health Insurance Service-Senior Cohort (2002-2013). Circ. J. 80, 2336–2342. 10.1253/circj.CJ-16-0692 27666598

[B75] Ibrahim S. S. (2008). Protective effect of hesperidin, a citrus bioflavonoid, on diabetes-induced brain damage in rats. J. Appl. Sci. Res. 4, 84–95.

[B76] International Diabetes Federation (2006). The IDF consensus worldwide definition of the METABOLIC SYNDROME, 2006. Availble online at: http://www.idf.org/e-library/consensus-statements/60-idfconsensus-worldwide-definitionof-the-metabolic-syndrome.

[B77] Jayaraman A. Pike C. J. (2014). Alzheimer's disease and type 2 diabetes: multiple mechanisms contribute to interactions. Curr. Diabetes Rep. 14:476. 10.1007/s11892-014-0476-2 24526623 PMC3985543

[B78] Kahn B. B. Flier J. S. (2000). Obesity and insulin resistance. J. Clin. Invest. 106, 473–481. 10.1172/JCI10842 10953022 PMC380258

[B79] Kaneto H. Fujii J. Myint T. Miyazawa N. Islam K. N. Kawasaki Y.. (1996). Reducing sugars trigger oxidative modification and apoptosis in pancreatic beta-cells by provoking oxidative stress through the glycation reaction. Biochem. J. 320, 855–863. 10.1042/bj3200855 9003372 PMC1218007

[B80] Kaneto H. Kajimoto Y. Fujitani Y. Matsuoka T. Sakamoto K. Matsuhisa M.. (1999). Oxidative stress induces p21 expression in pancreatic islet cells: possible implication in beta-cell dysfunction. Diabetologia 42, 1093–1097. 10.1007/s001250051276 10447521

[B81] Kaur J. (2014). A comprehensive review on metabolic syndrome. Cardiol. Res. Pract. 2014:943162. 10.1155/2014/943162 24711954 PMC3966331

[B82] Kawahito S. Kitahata H. Oshita S. (2009). Problems associated with glucose toxicity: role of hyperglycemia-induced oxidative stress. World J. Gastroenterol. 15, 4137–4142. 10.3748/wjg.15.4137 19725147 PMC2738809

[B83] Kawai T. Takei I. Tokui M. Funae O. Miyamoto K. Tabata M. (2010). Effects of epalrestat, an aldose reductase inhibitor, on diabetic peripheral neuropathy in patients with type 2 diabetes, in relation to suppression of Nε-carboxymethyl lysine. J. Diabetes Complicat. 24, 424–432. 10.1016/j.jdiacomp.2008.10.005 19716319

[B84] Kendall D. M. Kim D. Maggs D. (2006). Incretin mimetics and dipeptidyl peptidase-IV inhibitors: a review of emerging therapies for type 2 diabetes. Diabetes Technol. Ther. 8, 385–396 10.1089/dia.2006.8.385 16800760

[B85] Kim B. Feldman E. L. (2015). Insulin resistance as a key link for the increased risk of cognitive impairment in the metabolic syndrome. Exp. Mol. Med. 47:e149. 10.1038/emm.2015.3 25766618 PMC4351418

[B86] Kleinridders A. Ferris H. A. Cai W. Kahn C. R. (2014). Insulin action in brain regulates systemic metabolism and brain function. Diabetes 63, 2232–2243. 10.2337/db14-0568 24931034 PMC4066341

[B87] Koenig A. M. Mechanic-Hamilton D. Xie S. X. Combs M. F. Cappola A. R. Xie L.. (2017). Effects of the insulin sensitizer metformin in Alzheimer Disease: pilot data from a randomized Placebo-controlled Crossover Study. Alzheimer Dis. Assoc. Disord. 31, 107–113. 10.1097/WAD.0000000000000202 28538088 PMC5476214

[B88] Kong C. M. Lee X. W. Wang X. (2013). Telomere shortening in human diseases. FEBS J. 280, 3180–3193. 10.1111/febs.12326 23647631

[B89] Krentz A. Patel M. Bailey C. (2008). New drugs for type 2 diabetes mellitus: what is their place in therapy? Drugs 68:2131–2162. 10.2165/00003495-200868150-00005 18840004

[B90] Lam T. K. T. Schwartz G. J. Rossetti L. (2005). Hypothalamic sensing of fatty acids. Nat. Neurosci. 8, 579–584. 10.1038/nn1456 15856066

[B91] Li G. Mayer C. L. Morelli D. Millard S. P. Raskind W. H. Petrie E. C.. (2017). Effect of simvastatin on CSF Alzheimer disease biomarkers in cognitively normal adults. Neurology 89, 1251–1255. 10.1212/WNL.0000000000004392 28821686 PMC5606918

[B92] Li H. Horke S. Förstermann U. (2013). Oxidative stress in vascular disease and its pharmacological prevention. Trends Pharmacol. Sci. 34, 313–319. 10.1016/j.tips.2013.03.007 23608227

[B93] Lukačínová A. MojŽiš J. Benačka R. Racz O. Nistiar F. (2008). Structure-activity relationships of preventive effects of flavonoids in alloxan-induced diabetes mellitus in rats. J. Anim. Feed Sci. 17, 411–421. 10.22358/jafs/66635/2008

[B94] Lumeng C. N. Saltiel A. R. (2011). Inflammatory links between obesity and metabolic disease. J. Clin. Invest. 121, 2111–2117. 10.1172/JCI57132 21633179 PMC3104776

[B95] Maher P. Dargusch R. Ehren J. L. Okada S. Sharma K. Schubert D. (2011). Fisetin lowers methylglyoxal dependent protein glycation and limits the complications of diabetes. PLoS ONE 6:e21226. 10.1371/journal.pone.0021226 21738623 PMC3124487

[B96] Makar T. K. Rimpel-Lamhaouar K. Abraham D. G. Gokhale V. S. Cooper A. J. (1995). Antioxidant defense systems in the brains of type II diabetic mice. J. Neurochem. 65, 287–291. 10.1046/j.1471-4159.1995.65010287.x 7790873

[B97] Mamedov M. N. Shishkova V. N. (2007). Perspectives of the use of antihyperglycemic preparations in patients with metabolic syndrome and prediabetes. Kardiologiia 47, 88–93. 18260885

[B98] Marcano Torres M. (2004). Neuroprotección en enfermedad cerebrovascular. Gac Méd Caracas 112, 3–13.

[B99] Marchesi V. T. (2014). Alzheimer's disease and CADASIL are heritable, adult-onset dementias that both involve damaged small blood vessels. Cell. Mol. Life Sci. 71, 949–955. 10.1007/s00018-013-1542-7 24378989 PMC11113885

[B100] Maritim A. C. Sanders R. A. Watkins J. B. III. (2003). Diabetes, oxidative stress, and antioxidants: a review. J. Biochem. Mol. Toxicol. 17, 24–38. 10.1002/jbt.10058 12616644

[B101] Mattson M. P. Camandola S. (2001). NF-κB in neuronal plasticity and neurodegenerative disorders. J. Clin. Inv. 107, 247–254. 10.1172/JCI11916 11160145 PMC199201

[B102] Mayeux R. Stern Y. (2012). Epidemiology of Alzheimer diasease. Cold Spring Harb. Perspect. Med. 2:a006239. 10.1038/nrneurol.2011.2 22908189 PMC3405821

[B103] Medzhito R. Horng T. (2009). Transcriptional control of the inflammatory response. Nat. Rev. Immunology 9, 692–703. 10.1038/nri2634 19859064

[B104] Meier B. Radeke H. H. Selle S. Raspe H. H. Sies H. Resch K.. (1990). Human fibroblasts release reactive oxygen species in response to treatment with synovial fluids from patients suffering from arthritis. Free Radic. Res. Commun. 8, 149–160. 10.3109/10715769009087988 2158476

[B105] Meier B. Radeke H. H. Selle S. Younes M. Sies H. Resch K. (1989). Human fibroblasts release reactive oxygen species in response to interleukin-1 or tumour necrosis factor-α. Biochem. J. 263, 539–545. 2556998 10.1042/bj2630539 PMC1133461

[B106] Meier J. Nauck M. (2006). Incretins and the development of type 2 diabetes. Curr. Diab. Rep. 6, 194–201. 10.1007/s11892-006-0034-7 16898571

[B107] Meister B. (2007). Neurotransmitters in key neurons of the hypothalamus that regulate feeding behavior and body weight. Physiol. Behav. 92, 263–271. 10.1016/j.physbeh.2007.05.021 17586536

[B108] Merad-Boudia M. Nicole A. Santiard-Baron D. Saillé C. Ceballos-Picot I. (1998). Mitochondrial impairment as an early event in the process of apoptosis induced by glutathione depletion in neuronal cells: relevance to Parkinson's disease. Biochem. Pharmacol. 56, 645–655. 9783733 10.1016/s0006-2952(97)00647-3

[B109] Mergenthaler P. Lindauer U. Dienel G. A. Meisel A. (2013). Sugar for the brain: the role of glucose in physiological and pathological brain function. Trends Neurosci. 36, 587–597. 10.1016/j.tins.2013.07.001 23968694 PMC3900881

[B110] Milei J. Otero-Losada M. Llambí H. G. Grana D. R. Suarez D. Azzato F.. (2011). Chronic cola drinking induces metabolic and cardiac alterations in rats. World J. Cardiol. 3, 111–116. 10.4330/wjc.v3.i4.111 21526048 PMC3082734

[B111] Miranda M. Muriach M. Almansa I. Arnal E. Messeguer A. Díaz-Llopis M.. (2007). CR-6 protects glutathione peroxidase activity in experimental diabetes. Free Radic. Biol. Med. 43, 1494–1498. 10.1016/j.freeradbiomed.2007.08.001 17964420

[B112] Mitchell R. N. (2012). Mecanismos de lesión celular, in Patología Estructural y Funcional, 8th Edn., ed Stanley L. R. (Amsterdam: Saunders Elsevier), 20–22.

[B113] Moreira P. I. Cardoso S. M. Pereira C. M. Santos M. S. Oliveira C. R. (2009). Mitochondria as a therapeutic target in Alzheimer's disease and diabetes. CNS Neurol. Disord. Drug Targ. 8, 492–511. 10.2174/187152709789824651 19702564

[B114] Morgan M. J. Liu Z. G. (2011). Crosstalk of reactive oxygen species and NF-kappaB signalling. Cell Res. 21, 103–115. 10.1038/cr.2010.178 21187859 PMC3193400

[B115] Muriach M. Bosch-Morell F. Alexander G. Blomhoff R. Barcia J. Arnal E.. (2006). Lutein effect on retina and hippocampus of diabetic mice. Free Radic. Biol. Med. 41, 979–988. 10.1016/j.freeradbiomed.2006.06.023 16934681

[B116] Nagamatsu M. Nickander K. K. Schmelzer J. D. Raya A. Wittrock D. A. Tritschler H.. (1995). Lipoic acid improves nerve blood flow, reduces oxidative stress, and improves distal nerve conduction in experimental diabetic neuropathy. Diabetes Care 18, 1160–1167. 7587852 10.2337/diacare.18.8.1160

[B117] Nakamura J. Kato K. Hamada Y. Nakayama M. Chaya S. Nakashima E.. (1999). A protein kinase C-β-selective inhibitor ameliorates neural dysfunction in streptozotocin-induced diabetic rats. Diabetes 48, 2090–2095. 10.2337/diabetes.48.10.2090 10512378

[B118] National Cholesterol Education Program (NCEP) (2001). Expert Panel on Detection, Evaluation, and Treatment of High Blood Cholesterol in Adults (Adult Treatment Panel III). Third Report of the National Cholesterol Education Program (NCEP) Expert Panel on Detection, Evaluation, and Treatment of High Blood Cholesterol in Adults (Adult treatment panel III) final Report Circulation. 3143–3421. 12485966

[B119] Negre-Salvayre A. Coatrieux C. Ingueneau C. (2008). Advanced lipid peroxidation end products in oxidative damage to proteins. Potential role in diseases and therapeutic prospects for the inhibitors. Br. J. Pharmacol. 153, 6–20. 10.1038/sj.bjp.0707395 17643134 PMC2199390

[B120] Nettleton J. A. Harnack L. J. Scrafford C. G. Mink P. J. Barraj L. M. Jacobd D. R. Jr. (2006). Dietary flavonoids and flavonoid-rich foods are not associated with risk of type 2 diabetes in postmenopausal women. J. Nutr. 136, 3039–3045. 10.1093/jn/136.12.3039 17116717 PMC3034085

[B121] Nguyen D. V. Shaw L. C. Grant M. (2012). B. Inflammation in the pathogenesis of microvascular complications in diabetes. Front. Endocrinol. 3, 170 10.3389/fendo.2012.00170 PMC3527746 23267348

[B122] Niedowicz D. M. Daleke D. L. (2005). The role of oxidative stress in diabetic complications. Cell Biochem. Biophys. 43, 289–330. 10.1385/CBB:43:2:289 16049352

[B123] Nijveldt R. J. van Nood E. van Hoorn D. E. C. Boelens P. G. Van Norren K. van Leeuwen P. A. (2001). Flavonoids: a review of probable mechanisms of action and potential applications. Am. J. Clin. Nutr. 74, 418–425. 10.1093/ajcn/74.4.418 11566638

[B124] Norcini M. Vivoli E. Galeotti N. Bianchi E. Bartolini A. Ghelardini C. (2009). Supraspinal role of protein kinase C in oxaliplatin-induced neuropathy in rat. Pain 146, 141–147. 10.1016/j.pain.2009.07.017 19683395

[B125] Obadia N. Lessa M. A. Daliry A. Silvares R. R. Gomes F. Tibiriçá E.. (2017). Cerebral microvascular dysfunction in metabolic syndrome is exacerbated by ischemia-reperfusion injury. BMC Neurosci. 18:67. 10.1186/s12868-017-0384-x 28886695 PMC5591496

[B126] Okin D. Medzhitov R. (2012). Evolution of inflammatory diseases. Curr. Biol. 22, R733–R740. 10.1016/j.cub.2012.07.029 22975004 PMC3601794

[B127] Orsini M. Nascimento O. J. M. Matta A. P. C. Reis C. H. M. de Souza O. G. Bastos V. H.. (2016). Revisiting the term neuroprotection in chronic and Degenerative diseases. Neurol. Int. 8:6311. 10.4081/ni.2016.6311 27127599 PMC4830365

[B128] Otero-Losada M. Cao G. González J. Muller A. Ottaviano G. Lillig C.. (2015). Functional and morphological changes in endocrine pancreas following cola drink consumption in rats. PLoS ONE 10:e0118700. 10.1371/journal.pone.0118700 25790473 PMC4366068

[B129] Otero-Losada M. Cao G. Mc Loughlin S. Rodríguez-Granillo G. Ottaviano G. Milei J. (2014). Rate of atherosclerosis progression in ApoE^−/−^ mice long after discontinuation of cola beverage drinking. PLoS ONE 9:e89838 10.1371/journal.pone.0089838 24670925 PMC3966732

[B130] Otero-Losada M. Gómez Llambí H. Ottaviano G. Cao G. Muller A. Azzato F.. (2016a). Cardiorenal involvement in metabolic syndrome induced by cola drinking in rats: proinflammatory cytokines and impaired antioxidative protection. Mediators Inflamm. 2016:5613056. 10.1155/2016/5613056 27340342 PMC4906210

[B131] Otero-Losada M. González J. Müller A. Ottaviano G. Cao G. Azzato F.. (2016b). Exercise ameliorates endocrine pancreas damage induced by chronic cola drinking in rats. PLoS ONE 11:e0155630. 10.1371/journal.pone.0155630 27192084 PMC4871573

[B132] Otero-Losada M. Vila S. Azzato F. Milei J. (2013). Antioxidants supplementation in elderly cardiovascular patients. Oxid. Med. Cell. Longev. 2013:408260. 10.1155/2013/408260 24489984 PMC3899745

[B133] Pacher P. Beckman J. S. Liaudet L. (2007). Nitric oxide and peroxynitrite in health and disease. Physiol. Rev. 87, 315–424. 10.1152/physrev.00029.2006 17237348 PMC2248324

[B134] Packer L. Kraemer K. Rimbach G. (2001). Molecular aspects of lipoic acid in the prevention of diabetes complications. Nutrition 17, 888–895. 10.1016/S0899-9007(01)00658-X 11684397

[B135] Pahl H. L. (1999). Activators and target genes of Rel /NF-kB transcription factors. Oncogene 18, 6853–6866. 10.1038/sj.onc.1203239 10602461

[B136] Pan X. Chen Z. Fei G. Pan S. Bao W. Ren S.. (2016). Long-term cognitive improvement after benfotiamine administration in patients with Alzheimer's disease. Neurosci. Bull. 32, 591–596. 10.1007/s12264-016-0067-0 27696179 PMC5567484

[B137] Passos J. F. Saretzki G. Ahmed S. Nelson G. Richter T. Peters H.. (2007). Mitochondrial dysfunction accounts for the stochastic heterogeneity in telomere-dependent senescence. PLoS Biol. 5:e110. 10.1371/journal.pbio.0050110 17472436 PMC1858712

[B138] Pescio S. (2001). Tratamiento farmacológico de la hipertensión arterial. Drug treatment of hypertension. Medwave 1:e1908 10.5867/medwave.2001.02.1908

[B139] Pitsavos C. Panagiotakos D. Weinem M. Stefanadis C. (2006). Diet, exercise and the metabolic syndrome. Rev. Diabetic Stud. 3, 118–126. 10.1900/RDS.2006.3.118 17487335 PMC1783583

[B140] Poderoso J. J. (2016). The evolution of the earth and its atmosphere, in Biochemistry of Oxidative Stress, Physiopathology and Clinical Aspects, Vol. 16, eds Gelpi R. J. Boveris A. Poderoso J. J. (Springer International Publishing), 13–22.

[B141] Powers A. C. D'Alessio D. (2012). Páncreas Endócrino y Farmacoterapia de la Diabetes Mellitus e Hipoglucemia, in Goodman and Gilman's (2012) Las Bases Farmacológicas de la Terapéutica, 12th edn, eds Brunton L. L. Lazo J. S. Parker K. L. (Mexico: Mc Graw Hill), 1237–1275.

[B142] Preet A. Gupta B. L. Siddiqui M. R. Yadava P. K. Baquer N. Z. (2005). Restoration of ultrastructural and biochemical changes in alloxan-induced diabetic rat sciatic nerve on treatment with Na3VO4 and Trigonella: a promising antidiabetic agent. Mol. Cell. Biochem. 278, 21–31. 10.1007/s11010-005-7815-1 16180085

[B143] Purkayastha S. Zhang G. Cai D. (2011). Uncoupling the mechanisms of obesity and hypertension by targeting hypothalamic IKK-β and NF-κ B. Nat. Med. 17, 883–887. 10.1038/nm.2372 21642978 PMC3134198

[B144] Rahman I. Biswas S. K. Kode A. (2006). Oxidant and antioxidant balance in the airways and airway diseases. Eur. J. Pharmacol. 533, 222–239. 10.1016/j.ejphar.2005.12.087 16500642

[B145] Ramirez M. A. Borja N. L. (2008). Epalrestat: an aldose reductase inhibitor for the treatment of diabetic neuropathy. Pharmacotherapy 28, 646–655. 10.1592/phco.28.5.646 18447661

[B146] Rask-Madsen C. Kahn C. R. (2012). Tissue-specific insulin signaling, metabolic syndrome, and Cardiovascular disease. Arterioscler. Thromb. Vasc. Biol. 32, 2052–2059. 10.1161/ATVBAHA.111.241919 22895666 PMC3511859

[B147] Reagan L. P. Magariños A. M. Yee D. K. Swzeda L. I. Van Bueren A. McCall A. L.. (2000). Oxidative stress and HNE conjugation of GLUT3 are increased in the hippocampus of diabetic rats subjected to stress. Brain Res. 862, 292–300. 10.1016/S0006-8993(00)02212-5 10799703

[B148] Révész D. Milaneschi Y. Verhoeven J. E. Lin J. Penninx B. W. (2015). Longitudinal associations between metabolic syndrome components and telomere shortening. J. Clin. Endocrinol. Metab. 100, 3050–3059. 10.1210/JC.2015-1995 26133009

[B149] Rivas-Chávez J. Gutiérrez-Villafuerte C. Rivas-Legua J. (2007). Pharmacological treatment and costs of uncomplicated arterial hypertension. Rev. Soc. Peru Med. Int. 20:139.

[B150] Roberts C. K. Sindhu K. K. (2009). Oxidative stress and metabolic syndrome. Life Sci. 84, 705–712. 10.1016/j.lfs.2009.02.026 19281826

[B151] Roberts R. A. Smith R. A. Safe S. Szabo C. Tjalkens R. B. Robertson F. M. (2010). Toxicological and pathophysiological roles of reactive oxygen and nitrogen species. Toxicology 276, 85–94. 10.1016/j.tox.2010.07.009 20643181 PMC8237863

[B152] Rochette L. Lorin J. Zeller M. Guilland J. C. Lorgis L. Cottin Y.. (2013). Nitric oxide synthase inhibition and oxidative stress in cardiovascular diseases: possible therapeutic targets? Pharmacol. Ther. 140, 239–257. 10.1016/j.pharmthera.2013.07.004 23859953

[B153] Rockville M. D. (2007). Eisenberg center at oregon health & science university. comparing oral medications for adults with type 2 diabetes: Clinician's Guide. 2007 Dec 5, in Comparative Effectiveness Review Summary Guides for Clinicians: Agency for Healthcare Research and Quality (US). 21938805

[B154] Rojas-Gutierrez E. Muñoz-Arenas G. Treviño S. Espinosa B. Chavez R. Rojas K.. (2017). Alzheimer's disease and metabolic syndrome: a link from oxidative stress and inflammation to neurodegeneration. Synapse.. [Epub ahead of print]. 10.1002/syn.21990 28650104

[B155] Rozycka A. Jagodzinski P. P. Kozubski W. Lianeri M. Dorszewska J. (2013). Homocysteine Level and Mechanisms of Injury in Parkinson's disease as related to MTHFR, MTR, and MTHFD1 genes polymorphisms and L-Dopa treatment. Curr. Genomics 14, 534–542. 10.2174/1389202914666131210210559 24532985 PMC3924248

[B156] Salah S. H. Abdou H. S. Abdel Rahim E. A. (2010). Modulatory effect of vitamins A, C and E mixtures against tefluthrin pesticide genotoxicity in rats. Pestic. Biochem. Physiol. 98:191–197. 10.1016/j.pestbp.2010.06.006

[B157] Sato T. Hanyu H. Hirao K. Kanetaka H. Sakurai H. Iwamoto T. (2011). Efficacy of PPAR-γ agonist pioglitazone in mild Alzheimer disease. Neurobiol. Aging 32, 1626–1633 10.1016/j.neurobiolaging.2009.10.009 19923038

[B158] Schemmel K. E. Padiyara R. S. d'Souza J. J. (2010). Aldose reductase inhibitors in the treatment of diabetic peripheral neuropathy: a review. J. Diabetes Complicat. 24, 354–360. 10.1016/j.jdiacomp.2009.07.005 19748287

[B159] Schenk S. Saberi M. Olefsky J. M. (2008). Insulin sensitivity: modulation by nutrients and inflammation. J. Clin. Invest. 118, 2992–3002. 10.1172/JCI34260 18769626 PMC2522344

[B160] Shakher J. Stevens M. J. (2011). Update on the management of diabetic polyneuropathies. Diabetes Metab. Syndr. Obesity 4, 289–305. 10.2147/DMSO.S11324 21887102 PMC3160854

[B161] Sharma S. R. Sharma N. (2008). Epalrestat, an aldose reductase inhibitor, in diabetic neuropathy: an Indian perspective. Ann. Indian Acad. Neurol. 11, 231–235. 10.4103/0972-2327.44558 19893679 PMC2771994

[B162] Shoelson S. E. Goldfine A. B. (2009). Getting away from glucose: fanning the flames of obesity-induced inflammation. Nat. Med. 15, 373–374. 10.1038/nm0409-373 19350009 PMC4097148

[B163] Shukla V. Shakya A. K. Perez-Pinzon M. A. Dave K. R. (2017). Cerebral ischemic damage in diabetes: an inflammatory perspective. J. Neuroinflam. 14:21. 10.1186/s12974-016-0774-5 28115020 PMC5260103

[B164] Siegel A. B. Zhu A. X. (2009). Metabolic syndrome and hepatocellular Carcinoma, two growing epidemics with a potential link. Cancer 115, 5651–5661. 10.1002/cncr.24687 19834957 PMC3397779

[B165] Sies H. Jones D. P. (2007). Oxidative stress, in Encyclopedia of Stress, 2nd edn, Vol. 3, ed Fink G. (Amsterdam: Elsevier), 45–48.

[B166] Sonoda J. Pei L. Evans R. M. (2008). Nuclear receptors: decoding metabolic disease. FEBS Lett. 582, 2–9. 10.1016/j.febslet.2007.11.016 18023286 PMC2254310

[B167] Soto M. E. van Kan G. A. Nourhashemi F. Gillette-Guyonnet S. Cesari M. Cantet C.. (2013). Angiotensin-converting enzyme inhibitors and Alzheimer's disease progression in older adults: results from the Réseau sur la Maladie d'Alzheimer Français cohort. J. Am. Geriatr. Soc. 61, 1482–1488. 10.1111/jgs.12415 24000874

[B168] Spychalowicz A. Wilk G. ‘Sliwa T. Ludew D. Guzik T. J. (2012). Novel therapeutic approaches in limiting oxidative stress and inflammation. Curr. Pharm. Biotechnol. 13, 2456–2466. 10.2174/1389201011208062456 22280420

[B169] Staels B. Dallongeville J. Auwerx J. Schoonjas K. Loitersdorf E. Fruchart J. C.. (1998). Mechanism of action of fibrates on lipid and lipoprotein metabolism. Circulation 98, 2088–2093. 10.1161/01.CIR.98.19.2088 9808609

[B170] Stiles B. L. (2009). PI-3-K and AKT: onto the mitochondria. Adv. Drug Deliv. Rev. 61, 1276–1282. 10.1016/j.addr.2009.07.017 19720099

[B171] Stirban A. Negrean M. Stratmann B. Götting C. Salomon J. Kleesiek K.. (2007). Adiponectin decreases postprandially following a heat-processed meal in individuals with type 2 diabetes: an effect prevented by benfotiamine and cooking method. Diabetes Care 30, 2514–2516. 10.2337/dc07-0302 17630265

[B172] Suresh Kumar J. S. Menon V. P. (1993). Effect of diabetes on levels of lipid peroxides and glycolipids in rat brain. Metab. Clin. Exp. 402, 1435–1439. 10.1016/0026-0495(93)90195-T 8231839

[B173] Sweitzer N. K. (2003). What is an angiotensin converting enzyme inhibitor? Circulation 108:e16. 10.1161/01.CIR.0000075957.16003.07 12876137

[B174] Tankova T. Cherninkova S. Koev D. (2005). Treatment for diabetic mononeuropathy with α-lipoic acid. Int. J. Clin. Pract. 59, 645–650. 10.1111/j.1742-1241.2005.00452.x 15924591

[B175] Thompson P. D. Buchner D. Pina I. L. Balady G. J. Williams M. A. Marcus B. H.. (2003). Exercise and physical activity in the prevention and treatment of atherosclerotic cardiovascular disease. Circulation 107, 3109–3116. 10.1161/01.CIR.0000075572.40158.77 12821592

[B176] Tonkin A. Byrnes A. (2014). Treatment of dyslipidemia. F1000 Prime Rep. 6:17. 10.12703/P6-17 24669298 PMC3944745

[B177] Ulusu N. N. Sahilli M. Avci A. Canbolat O. Ozansoy G. Ari N.. (2003). Pentose phosphate pathway, glutathione-dependent enzymes and antioxidant defense during oxidative stress in diabetic rodent brain and peripheral organs: effects of stobadine and vitamin E. Neurochem. Res. 28, 815–823. 10.1023/A:1023202805255 12718433

[B178] Urios P. Grigorova-Borsos A. M. Sternberg M. (2007). Aspirin inhibits the formation of pentosidine, a cross-linking advanced glycation end product, in collagen. Diabetes Res. Clin. Pract. 77, 337–340. 10.1016/j.diabres.2006.12.024 17383766

[B179] Valko M. Leibfritz D. Moncol J. Cronin M. T. Mazur M. Telsar J. (2007). Free radicals and antioxidants in normal physiological functions and human disease. Int. J. Biochem. Cell Biol. 39, 44–84. 10.1016/j.biocel.2006.07.001 16978905

[B180] Vallianou N. Evangelopoulos A. Koutalas P. (2009). Alpha-lipoic acid and diabetic neuropathy. Rev. Diabetic Stud. 6, 230–236. 10.1900/RDS.2009.6.230 20043035 PMC2836194

[B181] Venkatesan R. Ji E. Kim S. Y. (2015). Phytochemicals that regulate neurodegenerative disease by targeting neurotrophins: a comprehensive review. Biomed Res. Int. 2015:814068. 10.1155/2015/814068 26075266 PMC4446472

[B182] Vieira M. N. N. Lima-Filho R. A. S. De Felice F. G. (2017). Connecting Alzheimer's disease to diabetes: underlying mechanisms and potential therapeutic targets. Neuropharmacology. [Epub ahead of print]. 10.1016/j.neuropharm.2017.11.014 29129775

[B183] Vincent A. M. Brownlee M. Russell J. W. (2002). Oxidative stress and programmed cell death in diabetic neuropathy. Ann. N. Y. Acad. Sci. 959, 368–383. 10.1111/j.1749-6632.2002.tb02108.x 11976211

[B184] Vinik A. I. Bril V. Kempler P. Litchy W. J. Tesfaye S. Price K. L.. (2005). Treatment of symptomatic diabetic peripheral neuropathy with the protein kinase C β-inhibitor ruboxistaurin mesylate during a 1-year, randomized, placebo-controlled, double-blind clinical trial. Clin. Ther. 27, 1164–1180. 10.1016/j.clinthera.2005.08.001 16199243

[B185] Vlckova V. Cornelius V. Kasliwal R. Wilton L. Shakir S. A. (2009). Hypoglycemia with oral antidiabetic drugs: results from prescription-event monitoring cohorts of rosiglitazone, pioglitazone, nateglinide and repaglinide. Drug Saf. 32, 409–418. 10.2165/00002018-200932050-00004 19419235

[B186] Wang G. G. Lu X. H. Li W. Zhao X. Zhang C. (2011). Protective effects of luteolin on diabetic nephropathy in STZ-induced diabetic rats. Evid. Based Comp. Alternat. Med. 2011:323171. 10.1155/2011/323171 21584231 PMC3092543

[B187] Watson G. S. Cholerton B. A. Reger M. A. Baker L. D. Plymate S. R. Asthana S.. (2005). Preserved cognition in patients with early Alzheimer disease and amnestic mild cognitive impairment during treatment with rosiglitazone: a preliminary study. Am. J. Geriat. Psychiatry 13, 950–958. 10.1176/appi.ajgp.13.11.950 16286438

[B188] Xu Q. G. Li X. Q. Kotecha S. A. Cheng C. Sun H. S. Zochodne D. W. (2004). Insulin as an *in vivo* growth factor. Exp. Neurol. 188, 43–51. 10.1016/j.expneurol.2004.03.008 15191801

[B189] Yagihashi S. Yamagishi S. I. Wada R. I. Baba M. Hohman T. C. Yabe-Nishimura C.. (2001). Neuropathy in diabetic mice overexpressing human aldose reductase and effects of aldose reductase inhibitor. Brain 124, 2448–2458. 10.1093/brain/124.12.2448 11701599

[B190] Yan H. Guo Y. Zhang J. Ding Z. Ha W. Harding J. J. (2008). Effect of carnosine, aminoguanidine, and aspirin drops on the prevention of cataracts in diabetic rats. Mol. Vis. 14, 2282–2291. 19081783 PMC2600521

[B191] Zárate A. Basurto L. Saucedo R. Hernandez-Valencia M. (2010). Guía para seleccionar el tratamiento farmacológico en diabetes 2. Rev. Med. Inst. Mex. Seguro Soc. 48, 293–296. 21192901

[B192] Zhang W. Xin L. Lu Y. (2017). Integrative analysis to identify common genetic markers of metabolic syndrome, dementia, and diabetes. Med. Sci. Monit. 23, 5885–5891. 10.12659/MSM.905521 29229897 PMC5737114

[B193] Zhang X. Xu L. He D. (2013). Endoplasmic reticulum stress-mediated hippocampal neuron apoptosis involved in diabetic cognitive impairment. Biomed Res. Int. 2013:924327. 10.1155/2013/924327 23710464 PMC3655482

[B194] Zhang X. Zhang G. Zhang H. Karin M. Bai H. Cai D. (2008). Hypothalamic IKKbeta /NF-kappaB and ER stress link overnutrition to energy imbalance and obesity. Cell 135, 61–73. 10.1016/j.cell.2008.07.043 18854155 PMC2586330

[B195] Zhao W. Q. Alkon D. L. (2001). Role of insulin and insulin receptor in learning and memory. Mol. Cell. Endocrinol. 177, 125–134. 10.1016/S0303-7207(01)00455-5 11377828

[B196] Ziegler D. Ametov A. Barinov A. Dyck P. J. Gurieva I. Low P. A.. (2006). Oral treatment with α-lipoic acid improves symptomatic diabetic polyneuropathy. Diabetes Care 29, 2365–2370. 10.2337/dc06-1216 17065669

[B197] Ziegler D. Low P. A. Litchy W. J. Boulton A. J. Vinik A. I. Freeman R.. (2011). Efficacy and safety of antioxidant treatment with α-lipoic acid over 4 years in diabetic polyneuropathy: the NATHAN 1 trial. Diabetes Care 34, 2054–2060. 10.2337/dc11-0503 21775755 PMC3161301

[B198] Ziegler D. Sohr C. G. H. Nourooz-Zadeh J. (2004). Oxidative stress and antioxidant defense in relation to the severity of diabetic polyneuropathy and cardiovascular autonomic neuropathy. Diabetes Care 27, 2178–2183. 10.2337/diacare.27.9.2178 15333481

[B199] Zlokovic B. V. (2008). New therapeutic targets in the neurovascular pathway in Alzheimer's disease. Neurotherapeutics 5, 409–414. 10.1016/j.nurt.2008.05.011 18625452 PMC2536515

